# Barriers to and Facilitators of Using Remote Measurement Technology in the Long-Term Monitoring of Individuals With ADHD: Interview Study

**DOI:** 10.2196/44126

**Published:** 2023-06-30

**Authors:** Hayley Denyer, Qigang Deng, Abimbola Adanijo, Philip Asherson, Andrea Bilbow, Amos Folarin, Madeleine J Groom, Chris Hollis, Til Wykes, Richard JB Dobson, Jonna Kuntsi, Sara Simblett

**Affiliations:** 1 Social, Genetic and Developmental Psychiatry Centre Institute of Psychiatry, Psychology and Neuroscience King's College London London United Kingdom; 2 South London and Maudsley NHS Foundation Trust London United Kingdom; 3 Attention Deficit Disorder Information and Support Service (ADDISS) Edgware, Middlesex United Kingdom; 4 The Department of Biostatistics and Health Informatics Institute of Psychiatry, Psychology and Neuroscience King’s College London London United Kingdom; 5 Institute of Health Informatics University College London London United Kingdom; 6 NIHR Biomedical Research Centre at South London and Maudsley NHS Foundation Trust and King’s College London London United Kingdom; 7 Health Data Research UK London University College London London United Kingdom; 8 NIHR Biomedical Research Centre at University College London Hospitals NHS Foundation Trust London United Kingdom; 9 School of Medicine, Mental Health & Clinical Neurosciences Institute of Mental Health University of Nottingham Nottingham United Kingdom; 10 NIHR MindTech Healthcare Technology Co-operative Institute of Mental Health University of Nottingham Nottingham United Kingdom; 11 Department of Psychology Institute of Psychiatry, Psychology and Neuroscience King's College London London United Kingdom

**Keywords:** attention-deficit/hyperactivity disorder, ADHD, remote measurement technology, engagement, barriers and facilitators, qualitative analysis, mobile phone

## Abstract

**Background:**

Remote measurement technology (RMT) has the potential to address current research and clinical challenges of attention-deficit/hyperactivity disorder (ADHD) symptoms and its co-occurring mental health problems. Despite research using RMT already being successfully applied to other populations, adherence and attrition are potential obstacles when applying RMT to a disorder such as ADHD. Hypothetical views and attitudes toward using RMT in a population with ADHD have previously been explored; however, to our knowledge, there is no previous research that has used qualitative methods to understand the barriers to and facilitators of using RMT in individuals with ADHD following participation in a remote monitoring period.

**Objective:**

We aimed to evaluate the barriers to and facilitators of using RMT in individuals with ADHD compared with a group of people who did not have a diagnosis of ADHD. We also aimed to explore participants’ views on using RMT for 1 or 2 years in future studies.

**Methods:**

In total, 20 individuals with ADHD and 20 individuals without ADHD were followed up for 10 weeks using RMT that involved active (questionnaires and cognitive tasks) and passive (smartphone sensors and wearable devices) monitoring; 10 adolescents and adults with ADHD and 12 individuals in a comparison group completed semistructured qualitative interviews at the end of the study period. The interviews focused on potential barriers to and facilitators of using RMT in adults with ADHD. A framework methodology was used to explore the data qualitatively.

**Results:**

Barriers to and facilitators of using RMT were categorized as *health-related*, *user-related,* and *technology*-*related* factors across both participant groups. When comparing themes that emerged across the participant groups, both individuals with and without ADHD experienced similar barriers and facilitators in using RMT. The participants agreed that RMT can provide useful objective data. However, slight differences between the participant groups were identified as barriers to RMT across all major themes. Individuals with ADHD described the impact that their ADHD symptoms had on participating (*health-related* theme), commented on the perceived cost of completing the cognitive tasks (*user-related* theme), and described more technical challenges (*technology-related* theme) than individuals without ADHD*.* Hypothetical views on future studies using RMT in individuals with ADHD for 1 or 2 years were positive.

**Conclusions:**

Individuals with ADHD agreed that RMT, which uses repeated measurements with ongoing active and passive monitoring, can provide useful objective data. Although themes overlapped with previous research on barriers to and facilitators of engagement with RMT (eg, depression and epilepsy) and with a comparison group, there are unique considerations for people with ADHD, for example, understanding the impact that ADHD symptoms may have on engaging with RMT. Researchers need to continue working with people with ADHD to develop future RMT studies for longer periods.

## Introduction

Attention-deficit/hyperactivity disorder (ADHD) is a common psychiatric disorder, with a prevalence of 2.5% among adults [1]. ADHD is diagnosed based on impairing levels of inattentive, hyperactive, and impulsive behaviors [2], but most adolescents and adults with ADHD present with additional co-occurring mental health problems manifesting in anxiety, depression, and poor sleep [1,3,4]. Although individual long-term outcomes are highly variable, adult ADHD has been associated with an increased risk of detrimental outcomes, including educational and occupational difficulties and antisocial behavior [3].

Individuals with ADHD are often assessed in a clinic or research laboratory setting, providing a snapshot of the symptoms and impairments they experience. Recent developments in remote measurement technology (RMT; including smartphone apps and wearable devices) offer new opportunities to address current research and clinical challenges [5,6]. For research, a remote ADHD assessment and monitoring battery will enable detailed, frequent, long-term, and real-world data collection on the clinical symptoms of ADHD and co-occurring disorders, functional and cognitive impairments, and health behaviors (such as exercise and sleep) in large sample sizes [5,7]. Longer term, an evidence-based remote ADHD assessment and monitoring battery also has the potential to transform clinical practice by offering the clinician easy and time-saving access to frequent detailed data on symptoms and impairments [5,8]. We have recently developed a new RMT system, the ADHD Remote Technology (ART) system, for adolescents and adults (aged ≥16 years) with ADHD that incorporates active (questionnaires and cognitive tasks) and passive (smartphone apps and a wearable device) monitoring.

Research using RMT has been successfully applied to many populations [9-11]. Despite participants and clinicians often reporting positively on the use of RMT [6,12-16], adherence and attrition are potential obstacles when applying RMT to clinical populations [11]. Individuals with ADHD often display difficulties with organization, following through on instructions, remembering daily tasks, and maintaining attention on difficult or boring tasks [2]. Although RMT allows for continuous, real-time data collection, it also requires frequent interaction and engagement from the participant, such as regular charging of devices and troubleshooting of technical malfunctions [17]. A substantial proportion of missing data or high dropout rates could result in the loss of statistical power and concerns about possible bias.

Hypothetical views and attitudes toward RMT in a population with ADHD have been explored previously [18]. Focus groups involving individuals with ADHD were positive about using RMT in clinical practice [18]. Despite positive reports, the study relied on theoretical scenarios, so we were unable to generalize these findings to the actual implementation of RMT. To our knowledge, no previous study has used qualitative methods to understand the end-point acceptability of RMT in individuals with ADHD. A strength of this study is that the individuals with ADHD all participated in the ART pilot study, which involved a 10-week remote monitoring period before a discussion on the barriers to and facilitators of RMT; thus, all participants had direct experience of using remote monitoring measures, including smartphone apps and wearable devices. The addition of a comparison group is also a novel approach to qualitative feedback on RMT. By including a comparison group, we were able to draw comparisons between individuals with ADHD and those without ADHD. The purpose of this study was to evaluate the barriers to and facilitators of using RMT in individuals with ADHD, compared with a group of people who did not have a diagnosis of ADHD. We also aimed to explore participants’ views on the use of RMT in future studies with a longer duration.

## Methods

### Design

The ART pilot study was an observational study involving a 10-week RMT study period, using active (questionnaires and cognitive tasks) and passive (wearable devices and smartphones) monitoring. The ART pilot study involved 2 participant groups: adolescents and adults with ADHD and a comparison group (individuals without ADHD). Semistructured interviews were completed after participating in the study, following a topic guide on the barriers to and facilitators of using RMT in individuals with ADHD and in those without ADHD.

### Participants

Participants were eligible to participate in the ART pilot study if they were aged between 16 and 60 years, able to provide informed consent, fluent in English, and willing to wear a wearable device and use an Android phone during the 10-week remote monitoring period. Participants with ADHD were eligible if they met the Diagnostic and Statistical Manual of Mental Disorders, Fifth Edition, criteria for ADHD, and participants in the comparison group were eligible if they did not reach the threshold for ADHD on the Barkley symptom and impairment scale [19]. Participants in the ART pilot study were recruited from previous studies (where they had indicated that they were willing to be contacted regarding future research studies), via the Attention Deficit Disorder Information and Support Service, social media, and King’s Volunteer circular and on the “Call for Participants” website.

### Procedure

**Figure 1:**
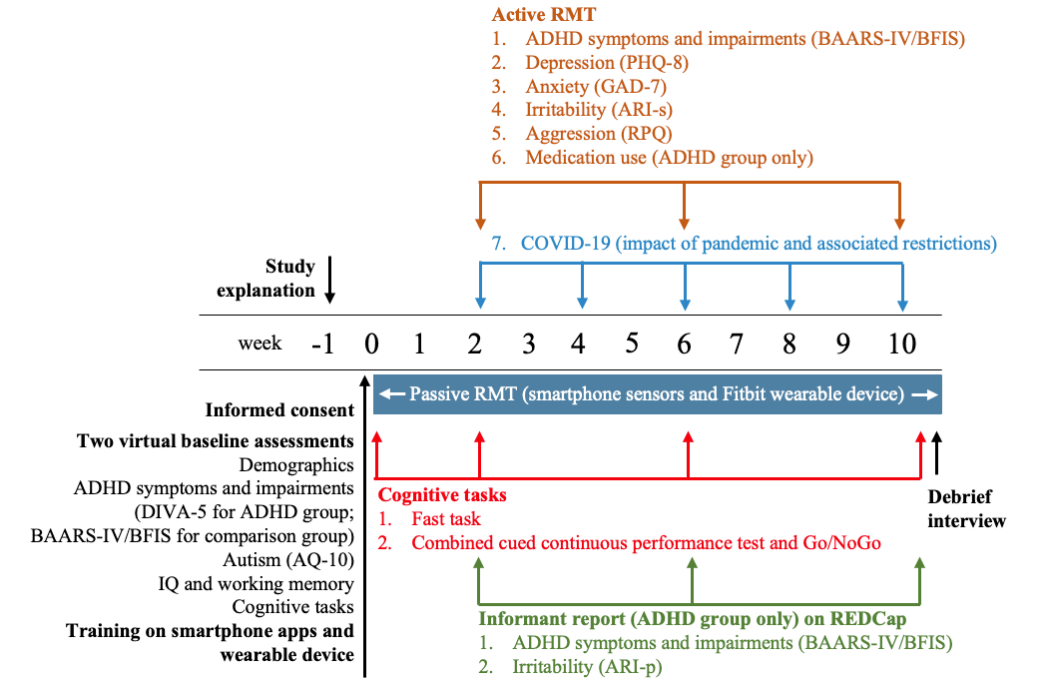
ADHD Remote Technology pilot study design. ADHD: attention-deficit/hyperactivity disorder; AQ-10: autism spectrum quotient; ARI-p: Affective Reactivity Index-parent-report; ARI-s: Affective Reactivity Index- self report; BAARS-IV/BFIS: Barkley Adult ADHD Rating Scale—IV/Barkley Functional Impairment Scale; DIVA-5: Diagnostic Interview for Adult ADHD; GAD-7: Generalized Anxiety Disorder-7; PHQ-8: Patient Health Questionnaire-8; REDCap: Research Electronic Data Capture; RPQ: Reactive-Proactive Aggression Questionnaire; RMT: remote measurement technology.

Participants attended 2 remote baseline sessions with a research worker using Microsoft Teams. The first remote baseline session with the participants with ADHD included the administration of the Diagnostic Interview for ADHD in adults [20] to confirm ADHD diagnosis and cognitive and self-report measures, which are beyond the scope of this study (Figure 1). Participants without ADHD in the comparison group were assessed in the same way, except that instead of the full ADHD diagnostic interview, they completed the ADHD symptom and impairment questionnaire [19].

The second session was administered once participants had received their wearable device (Fitbit Charge 3) and Android smartphone via post (if they were not using their own compatible Android device) approximately a week after the first session. It included training on the use of wearable devices and on the smartphone Passive and Active Apps. The participants also received a leaflet summarizing key information (Participant Technology User Guide) and researcher contact details for future reference.

All participants then participated in a 10-week remote monitoring period using a wearable device and smartphone. RMT incorporated active (questionnaires and cognitive tasks) and passive (wearable device and smartphone sensors) monitoring. Active monitoring involved the participants completing clinical symptom questionnaires on the smartphone Active App and cognitive tasks on their home PC or laptop during weeks 2, 6, and 10 (Figure 1). Participants completed 2 computer-based cognitive tasks that were sensitive to differences between individuals with ADHD and those without ADHD [21]. Passive App monitoring data (data that do not require any conscious engagement from the participant) were collected continuously on a 24/7 basis through a smartphone and a Fitbit device (Fitbit Charge 3). The smartphone Passive App collected data on a range of measures, such as ambient noise, ambient light, phone use information (eg, which apps were used and how long and when the phone was unlocked), and relative location. In this study, we used participants’ personal Android devices where available and provided a participant with a Motorola G7 Play or G7 Power if they had an iPhone or did not have a smartphone. Participants were asked to wear the Fitbit on their nondominant hand.

At the end of the 10-week remote monitoring period, participants were invited to participate in an optional “debrief” session, which included a qualitative interview to investigate their experiences of participating. The data collected during the interviews formed the basis of this study. The interviews, which lasted 25 to 50 minutes, were conducted by 2 research workers (QD and HD) and focused on potential barriers to and facilitators of RMT using a semistructured framework that covers participant experience, enrolling into the study and support, working the study into daily life, experience of using the devices, data privacy and sharing, and future uses.

### Ethics Approval, Informed Consent, and Participation

The interview and study procedures were approved by the North East—Tyne and Wear South Research Ethics Committee (20/NE/0034; IRAS ID: 278126). Informed consent was obtained from all participants before the assessments started. To maintain participant anonymity, their data were pseudonymized. The participants’ names were replaced with a code. Data collected from apps, wearable devices, and interviews were only associated with this code and stored separately from any personally identifiable information. Participants were compensated £30 (US $37) after the completion of the baseline sessions, £20 (US $25) after the first remote active monitoring follow-up (end of week 5), and a further £50 (US $62) at the study end point (end of week 10). The participants did not receive additional compensation for completing the debrief interviews.

### Data Analysis

Descriptive statistics were calculated for gender, age, and ethnicity. All interviews were audio recorded and transcribed verbatim. A framework methodology was used to qualitatively explore the data [12]: (1) transcription, (2) familiarization with the interviews, (3) coding by 2 researchers working independently (HD and AA) using the qualitative software program NVivo Software (version 2020), (4) developing a working analytical framework (HD and AA), (5) applying the analytical framework (HD), (6) charting data into a framework matrix (HD), and (7) interpreting the data (HD and SS). The analytical framework was built on barriers to and facilitators of engagement with RMT identified in a recent systematic review [9]. We then identified themes that deviated across participant groups using the charted framework matrix.

The framework methodology is suitable for the analysis of interview data, where it is desired to make comparisons within and between cases [22]. Percentages are used to compare the themes that deviated between individuals with ADHD and individuals without ADHD in a comparison group.

## Results

### Participants

In total, 20 individuals with ADHD and 20 individuals without ADHD in a comparison group participated in the ART pilot study (Table 1). Of the participants who participated in the ART pilot study, 22 participants (10 individuals with ADHD and 12 individuals in the comparison group) opted to complete audio recorded debrief interviews at the end of the study period. Of these participants, 91% (20/22) completed the debrief interview within 2 months of completing their remote monitoring period, and all participants completed the debrief interview within 6 months. Interviews were completed until data saturation, which refers to a point in the data collection process where the interviewer keeps hearing repeated information or when no new information is identified [23,24].

**Table 1:** Participant characteristics.

		ADHD^a^ Remote Technology pilot study			Debrief interviews	
		ADHD group (n=20)	Comparison group (n=20)	ADHD group (n=10)		Comparison group (n=12)
Gender (female), n (%)		15 (75)	15 (75)	8 (80)		11 (91)
Age (years), mean (SD)		27.49 (6.04)	27.79 (6.17)	28.03 (6.36)		27.75 (6.74)
**Ethnicity, n (%)**						
	Asian	1 (5)	3 (15)	0 (0)		3 (25)
	Black	1 (5)	0 (0)	1 (10)		0 (0)
	White	16 (80)	17 (85)	9 (90)		9 (75)
	Mixed or multiple ethnic groups	2 (10)	0 (0)	0 (0)		0 (0)

^a^ADHD: attention-deficit/hyperactivity disorder.

### Barriers to and Facilitators of RMT

Multimedia Appendix 1 presents the 3 themes of barriers to and facilitators of RMT that emerged across participant groups. These themes have been subdivided into health-related, user-related, and technology-related themes to guide the understanding of which aspects of the system we need to target to improve engagement.

Table 2 presents the fourth theme that is based on hypothetical views of what participants would want from future research.

**Table 2:** Hypothetical views on what participants would want from the study in the future.

Future studies					ADHD^a^ group (n=10), n (%)		Comparison group (n=12), n (%)		Total (n=22), n (%)
**Willingness**									
	Positive about using the system for 1 or 2 years			8 (80)		10 (83)		18 (82)	
**Sharing data with general practitioners**									
	**Acceptable**			5 (50)		11 (92)		16 (73)	
		Benefit their care		6 (60)		7 (58)		13 (59)	
		Access to objective data		2 (20)		4 (33)		6 (27)	
		Real-time access		2 (20)		1 (8)		3 (14)	
		Taking action		5 (50)		5 (43)		10 (45)	
	Questioned whether all RMT^b^ data are relevant			5 (50)		1 (8)		6 (27)	
	Choice			3 (30)		3 (25)		6 (27)	
**Improvements needed**									
	**Feedback**								
		Wanted more feedback		4 (40)		2 (17)		6 (27)	
		Visual feedback		4 (40)		5 (42)		9 (41)	
		Verbal feedback		2 (20)		1 (8)		3 (14)	
		Feedback on active data		5 (50)		3 (25)		8 (36)	
		Feedback on passive data		3 (30)		7 (58)		10 (45)	
		During study period		2 (20)		2 (17)		4 (18)	
		End of study		4 (40)		5 (42)		9 (41)	
	More information			4 (40)		1 (8)		5 (23)	
	**Passive monitoring**								
		Passive app syncing issues		3 (30)		5 (42)		8 (36)	
		Using own phone		3 (30)		2 (17)		5 (23)	
	**Cognitive tasks**								
		Shortening the length		5 (50)		2 (17)		7 (32)	
		Less frequent		3 (30)		2 (17)		5 (23)	

^a^ADHD: attention-deficit/hyperactivity disorder.

^b^RMT: remote measurement technology.

### Health-Related Theme

#### Insight

For participants with ADHD, RMT provided better insight into their health-related behaviors and lifestyle. For health-related behaviors, RMT (specifically the wearable device) provided better insights into their sleep and physical activity levels, and participants with ADHD described improvements in these health-related behaviors. In terms of lifestyle, RMT provided participants with ADHD better insight into their routines and phone use, and some participants also described an improvement in these areas. For example, 1 participant with ADHD explained as follows:

I’ve definitely found it helpful in sort of motivating me to keep a good routine and try and get my steps in every day. So, like exercise and health wise, it’s been really good.

ADHD03

Participants in the comparison group also agreed that RMT gave them better insight into their sleep and physical activity and noted improvements in these health-related behaviors:

Well, having the Fitbit data that’s very helpful...I’ve been using the Fitbit data actively to monitor my exercises or meditation, sleep quality, so that was very useful.

COMP21

#### Impact of ADHD Symptoms on RMT

Participants with ADHD commented on the impact of ADHD symptoms on taking part in the study. Participants with ADHD described difficulties with attention, forgetfulness, motivation to complete demanding tasks, and organization, which impacted their experiences in the study. Difficulties with forgetfulness included remembering to complete questionnaires and cognitive tasks and to charge the devices. One participant explained as follows:

I’m not really organised with things. Things happen at the spare of the moment...I’m not someone that can pre-think that my Fitbit’s gonna run out of charge, it would be like “oh, now it has now run out of charge.”

ADHD01

### User-Related Theme

#### Perceived Costs

Perceived costs relate to any aspects of RMT that made participation challenging or difficult. The most prominent perceived cost for participants with ADHD was the cognitive tasks. There were also some perceived costs relating to passive monitoring measures, such as charging the devices, adapting to the new study phone, and phone connectivity issues (eg, synchronizing data from the wearable device to the phone). The comparison group reported fewer personal costs.

#### Compatibility

There was a consensus among participants in both groups that the ART pilot study fit into their daily life. One participant explained as follows:

The overall experience was like very easy...I was just carrying on with my normal life.

ADHD04

Another participant described that they *“*could honestly just go about my daily life and participate, yeah, passively” (ADHD09). Despite the study blending into participants’ daily life, some participants with ADHD mentioned difficulty finding the time to complete the cognitive tasks in a quiet and distraction-free environment. This was also mentioned in the comparison group:

I did sometimes struggle to find like an hour that I could just block off and have no distractions and things, that was a bit more tricky.

COMP13

#### Intrinsic Value

There was agreement among participants with ADHD that the benefits of participating outweighed the costs of participating in the study. For example, 1 participant with ADHD explained as follows:

Oh no, definitely benefits outweigh, like there wasn’t really any cons, it was very simple and straightforward. But then like benefits, yup definitely more aware of my sleep and my exercise. So really, really outweighed the cons, definitely.

ADHD04

Participants in the comparison group also agreed that the benefits outweighed the costs, with 1 participant stating, “100%, like, it’s definitely worth taking part” (COMP16).

#### Technology Acceptance

All participants with and without ADHD commented on the acceptability and ease of collecting the passive data**,** with 1 participant explaining as follows:

Yeah, I mean that’s easier to be honest, um, as long as I know what data is being collected and it’s not particularly, um, kind of sensitive data, it’s not looking at kind of what’s going in and out of text message conversations, for example. Um, it’s easier to have that collected passively for me personally, rather than to actively remember to upload something.

COMP19

Participants explained that they accepted RMT measures as technology nowadays collects these data. Participants with and without ADHD mentioned that the study had been well explained and were reassured that the study had gone through the correct reviews, so they could trust that the study was ethical, that it would be safe for them to take part, and that their data would be kept anonymous.

#### Overall Experience

Overall, the participants’ experience using the smartphone and wearable device in the ART system was positive. Participants both with and without ADHD described the study as easy, enjoyable, and interesting. One participant with ADHD explained as follows:

You know ’cause it gave me a very new exciting toy to play with...and I mean it was very exciting. You know, the study wasn’t too long that it got boring, you know?

ADHD02

Participants with and without ADHD also noted that the study period went quickly and that the study was well organized.

Few participants with ADHD described the study as demanding or that it required a short-term adjustment period. However, more participants in the comparison group described the study as long, demanding, or requiring an adjustment period. For example, 1 participant in the comparison group explained as follows:

I guess it was, it was more of an intensive or like a like a longer study than ones I’ve done before.

COMP13

Despite these comments, these participants also reported that the overall experience of the study was positive and that the benefits outweighed any costs.

### Technology-Related Theme

#### Value in Gathering RMT Data

There was a consensus among participants that there was value in gathering RMT data. One participant with ADHD explained as follows:

I think it’s really great. Um, I think it really, sort of improves the ability to get accurate and up-to-date data. I think it’s great.

ADHD10

In general, participants described passive data as collecting objective measures of their health and that such data are more accurate than “manually record[ing] it yourself” (ADHD03).

Participants with and without ADHD commented that passive monitoring was better than manually providing the data, as it improved the accuracy, the amount of data collected, and the ability to observe behavioral patterns and to compare variables. A participant with ADHD commented as follows:

I mean, it certainly made it more accurate ’cause I’m very aware now that if I’d been filling out forms, they would not have been accurate at all because I had absolutely no awareness of how much sleep I was getting.

ADHD10

#### Convenience

Participants with ADHD expressed the view that the passive monitoring aspect made it easier for the participant to participate, for example:

With the passive monitoring so like when you’re monitoring sleep, the fact that you’re not kind of having to manually record it yourself, and it is done all automatically, I think that sort of, umm, I think if you were having to like manually record it and go to that effort, you would, I think it would have an influence on your, sort of, approach to kind of going to sleep and things like that. Whereas when it’s done passively, like you’re not having that kind of, um, that you’re not putting that input in yourself.

ADHD03

Participants in the comparison group also commented that passive monitoring made it easier for them to participate.

#### Intrusiveness

Cognitive tasks were described as the most intrusive aspect of technology-related barriers to RMT. All participants with ADHD described the cognitive tasks as tedious, for example:

Yeah, I’m not sure if there’s anything that can be done about it...but the tasks were really difficult...I’m not sure how like a neurotypical person would find it, but sort of sitting and looking at a screen with no stimulation other than it for close to an hour was really hard!

ADHD10

In general, participants with ADHD viewed cognitive tasks as being too long or commented that the frequency of completing the tasks was too often. The cognitive tasks were also described by the participants in the comparison group as tedious, the tasks were viewed as too long, and participants commented on the frequency of the tasks being too often.

Some participants expressed concerns over monitoring; for example, they had initial concerns that the research team would be monitoring their location. In total, 3 participants questioned the relevance of the aggression questionnaire they were asked to complete.

#### Usability

Participants described in detail the usability of the Active App, Fitbit wearable device, and study Android phone. For the Fitbit, participants with ADHD noted that it was comfortable; however, they reported some adherence difficulties and technical issues during the study period. Adherence difficulties included remembering to charge and remembering to wear the Fitbit again after charging or showering. Technical issues described by participants with ADHD were related to charging issues and syncing the Fitbit with the smartphone. Participants in the comparison group also commented on their comfort, adherence difficulties, and technical issues.

Technical challenges with the study Android phone were reported by individuals with ADHD; these included problems with the Passive App including synchronizing and transfer issues. Despite reporting some technical issues with the Passive App, participants with ADHD mentioned that the phone was easy to use and that they were able to use the phone normally. The questionnaires on the Active App were described as being easy to complete.

### Future Study Theme

#### Willingness

Participants were asked for their views on using the ART system in the longer term. Overall, participants with ADHD were positive about using the system for 1 or 2 years. One participant explained as follows:

Yeah, I think because it was so behind the scenes, I didn’t have to do anything. You were just sort of looking at what I’m doing on my normal day-to-day. Yeah, it wouldn’t bother me [to take part for] a year.

ADHD04

Participants in the comparison group also agreed with this; for example, 1 participant explained as follows:

Yes,...I wouldn’t find it like a very big strain to participate in the study if it was exactly as it was for a longer period of time.

COMP20

#### Sharing Data With General Practitioners

Participants were asked to consider how they would feel about RMT data being shared with the general practitioner (GP). There was some discrepancy among participants with ADHD regarding whether they would find it acceptable to share these data with GPs, whereas the comparison group agreed that this would be acceptable. In general, those who would be willing to share RMT data felt it could benefit the care they received, as it would provide GPs real-time access to objective data that they could then take action on. One participant with ADHD explained as follows:

I think that could be sort of quite beneficial ’cause then it would then help, say the GP or whoever, to understand a bit better exactly what’s going on...They can kind of see from the data...what you’re saying is, is how it is.

ADHD03

Those who would be less willing to share RMT data with GPs questioned whether all RMT data would be relevant to share with their GPs. Despite this, participants with ADHD emphasized they would find it more acceptable if they had a choice in what aspects of RMT would be shared, for example:

I would prefer to choose as some of it obviously, I would feel isn’t relevant to me and they don’t need to [know].

ADHD09

Only 1 participant in the comparison group expressed concerns about whether the data collected were relevant to their health care.

#### Improvements Needed

Participants with and without ADHD described improvements that could be made to the ART system for future long-term studies. This included additional feedback during the study period. Participants suggested that feedback should be given on both active and passive monitoring data in either a visual or verbal format, which would be shared both during and at the end of the study.

Some participants with ADHD suggested that providing more information would be beneficial for future studies, such as information on adapting to the Android phone, data use, video explanations, and leaflets for technology instructions. However, 1 participant with ADHD commented that the information sheets contain too much information and should be shortened.

The participants suggested changes specific to the active and passive monitoring aspects of ART system, including cognitive tasks (active monitoring) and the smartphone (passive monitoring). The participants with ADHD suggested two ways to improve the administration of the cognitive tasks: (1) shortening the length, for example, 1 participant explained, *“*anything that could be done, just like you know shorter but more frequent—that would’ve been great!*”* (ADHD10); and (2) less-frequent administration, for example, *“*Yeah, I think if you’re doing it for a much longer period, I would definitely say needs to be a longer gap between them*”* (ADHD03). Participants in the comparison group also agreed with these suggestions; for example, 1 participant in the comparison group explained as follows:

You know, maybe if you did a yearlong study, you maybe wouldn’t need to do [the cognitive tasks] quite so often.

COMP13

Improvements in passive monitoring aspects of the ART system were related to the study smartphone. Participants with ADHD suggested improving syncing issues of the Passive App and that it would be better to be able to use their own phone during the study period, instead of a study Android phone. Participants in the comparison group also made these suggestions.

### Differences Between Individuals With and Without ADHD

When comparing themes that emerged across the participant groups (Multimedia Appendix 1 and Table 2), individuals with and without ADHD experienced similar barriers to and facilitators of using RMT. However, slight differences between participant groups were identified across all 4 themes. In the health-related theme, all participants with ADHD identified the impact of ADHD symptoms as a potential barrier to RMT. Examples of the impact of ADHD symptoms included forgetfulness, disorganization, and difficulties with attention. In comparison, only 1 participant in the comparison group reported that forgetfulness was a potential barrier to RMT. Some participants with ADHD reported improvements in lifestyle; however, participants in the comparison group did not report this.

In the user-related theme, more individuals with ADHD noted that the cognitive tasks (active monitoring) were a perceived cost compared with the comparison group. This would be expected because of the demand of the cognitive tasks in individuals who have difficulties with attention to demanding tasks, that is, the cognitive tasks have been specifically designed to be challenging for individuals with ADHD in order for them to be informative about the differences between people with and without ADHD. Another perceived cost for individuals with ADHD was the charging of the devices, whereas participants in the comparison group did not mention this. In the overall experience minor theme, the negative subtheme highlighted some differences between individuals with and without ADHD. Compared with participants with ADHD, more participants in the comparison group described the study as long, demanding, or requiring an adjustment period.

In the technology-related theme, individuals with ADHD described more technical challenges related to the study Android phone, compared with those in the comparison group. In the future studies theme, there was disagreement among the individuals with ADHD about sharing RMT data with the GP, whereas participants in the comparison group found this acceptable.

## Discussion

### Principal Findings

This study builds on a growing number of RMT studies in clinical populations. Our findings provide novel insights into the barriers to and facilitators of using RMT in individuals with ADHD who have completed a remote monitoring study period that uses repeated measurements with ongoing active (eg, questionnaires and cognitive tasks) and passive (eg, smartphone sensors and wearable devices) monitoring. This study highlights the similarities and some, albeit few, differences between individuals with and without ADHD interpreted as barriers to and facilitators of RMT. The ART pilot study also shares similar methods and measures as the RMT in individuals with depression and epilepsy [25,26], and the topic guide and coding frame were adapted from a study that explored barriers to and facilitators of using RMT in individuals with depression [9], so comparisons can be drawn with other clinical populations.

### Insight Into Health-Related Behaviors

Individuals with ADHD, as well as the comparison group, commented on the benefits of using the wearable device, including them obtaining better insight and being able to observe improvements in their sleep and physical activity. Although these insights may have helped maintain participant engagement with the study, we note that using the devices to change behavior was not an aim in the ART pilot study (ie, the research team had emphasized that the participants should continue their behaviors and daily lives as normal). The ART pilot study involved a remote monitoring period of 10 weeks, and over a longer remote monitoring period, these effects may be reduced, as participants have longer to acclimatize to wearing and using the devices, so its novelty of receiving insight into health-related behaviors may reduce. Future research should consider the type of feedback provided by the wearable devices.

### Concerns Over Core ADHD Symptoms

Owing to individuals with ADHD often reporting difficulties with following through on instructions, remembering daily tasks, and maintaining attention on demanding tasks [2], we hypothesized that this could be a potential barrier when developing a remote measurement system for individuals with ADHD. All participants with ADHD spoke about the difficulties their ADHD symptoms had on participating, including difficulties with organization, motivation to complete demanding tasks, and forgetfulness. Symptoms such as forgetfulness have also been raised as a potential barrier to RMT in other psychiatric disorders (including depression) [9]. Only 1 individual from the comparison group commented that forgetfulness was a potential barrier to the ART system. It could be suggested that forgetfulness and other related ADHD symptoms may be a barrier to future RMT studies, and further support should be in place to help support individuals with ADHD in RMT studies. The ART system included reminders for questionnaire and cognitive task completions; however, future RMT studies with individuals with ADHD should go further, for example, by providing notifications to charge and replace the wearable device. We have already incorporated this consideration in our ongoing European Union–funded clinical study “ADHD Remote Technology Study of Cardiometabolic Risk Factors and Medication Adherence,” where reminders are set up to notify the participant after a prolonged period of not wearing the device and when their wearable device is fully charged [27].

### Cognitive Tasks

Barriers to using RMT in individuals with ADHD are mainly related to the perceived cost and intrusiveness of cognitive tasks (the frequency of administration and time taken to complete them). The tasks were completed during weeks 2, 6, and 10; this is the first study in which multiple measurements using cognitive tasks were collected in a 10-week period. Future studies should allow more time between the tasks to ensure participants are not fatigued and their continued participation in the study.

### Importance of Compatibility

Participants noted that the benefits of participating in an RMT study outweighed any costs. This finding is positive for future studies using RMT in individuals with ADHD. Despite initial concerns that using RMT with individuals with ADHD relies on the participant to engage with the technology and researchers, all participants with ADHD described the study as fitting into their daily lives. The compatibility of RMT was also described by all individuals in the comparison group. The importance of compatibility with one’s existing routine was also described in individuals with major depressive disorder [9].

### Collecting and Sharing of RMT Data

As RMT collects vast amounts of data on a variety of different measures, it is expected that concerns about data privacy may arise. Privacy concerns have also been raised in other RMT studies, including individuals with depression and epilepsy [9,28]. Although we observed some concerns over monitoring and adherence difficulties, participants explained that they were reassured, as the study was explained well, and they trusted that the research had undergone the right ethical checks. This is positive as the interest in RMT studies has gained momentum. Future RMT studies explicitly need to consider the importance of choice and control around data sharing when designing an RMT study.

Individuals with epilepsy have reported interest in using RMT as a method to remember and communicate with health care professionals [28]. Individuals with ADHD also described the potential use of presenting RMT data to their GP or health care professionals as a way of remembering or providing evidence about their ADHD symptoms or co-occurring health behaviors. In contrast, there were some concerns about sharing RMT data with their health care professionals. However, it is important to note that despite asking participants, “How would you feel about the information collected during the study going to your GP or healthcare provider?” those who questioned whether all RMT data would be relevant to their care only referred to GPs and did not refer to other health care professionals. Our findings may not apply to views of sharing data with other health care professionals, such as clinicians involved in medication titration, who may be in more frequent contact with individuals with ADHD and where it may benefit the care they receive if their clinical care team had access to frequent questionnaire completion or sleep behaviors.

### Future Research

Building on the barriers to and facilitators of RMT described by individuals with ADHD, future research using RMT should incorporate our suggestions for long-term monitoring of individuals with ADHD (Textbox 1). These recommendations have been considered at the individual and group levels. At an individual level, individuals with ADHD emphasized some privacy concerns and the importance for the individual to have a choice over the types of RMT data that are collected and then shared with health care professionals. Individuals with ADHD also seemed to report varying levels of technical issues and an adjustment period for the devices provided. The type of data (active or passive data), the format (visual or verbal), and the frequency (during or at the end of the study period) that feedback is provided should be tailored to the individual to help with continued engagement with RMT.

At a group level, individuals with ADHD were positive about using the ART system for 1 or 2 years. There was agreement that despite some technical issues, RMT collected objective data and had the potential to help provide evidence about their ADHD symptoms or co-occurring health behaviors to their GP or health care professionals. On the basis of the reports that individuals with ADHD felt their symptoms impacted their study experience, future research should incorporate ADHD-specific support from researchers to help with the core symptoms of ADHD (eg, reminders) to promote with adherence. There was consensus that future studies using cognitive tasks should be conducted less frequently.

**Researchers’ suggestions for future studies based on the barriers to and facilitators of using remote measurement technology (RMT).**

**Privacy concerns**

- Emphasis should be placed on the participants’ control over their data and their choice to share data with health care professionals.

**Technical issues**

- Technical issues will happen, but the overall consensus is that the smartphone apps and wearable devices allow for objective and accurate data collection.

**Adjustment period**

- Allow for an adjustment period while the participant becomes familiar with the new devices. In addition, provide self-help guides and videos to aid with the introduction of new devices

**Feedback**

- Feedback to individuals with attention-deficit/hyperactivity disorder should be personalized based on the types of RMT data that individual would like to see and in the form they would find most useful (eg, visual or verbal feedback).

**Adherence**

- Reminders throughout the study period, including to complete questionnaires, are vital for successful completion rates, but reminders should also be extended to reminding the participant to replace the wearable device after it is removed for charging or showering.

**Cognitive tasks**

- Fewer cognitive tasks during a study period with longer completion times

### Strengths and Limitations

The use of qualitative methods allows for an in-depth exploration and discussion of the potential barriers to and facilitators of using RMT, such as smartphone apps and wearable devices, for a remote assessment and monitoring system that can be applied to both research and clinical long-term monitoring of symptoms, impairments, and health-related behaviors associated with ADHD.

Previous qualitative research has relied on hypothetical views of using RMT [18,28,29]. A strength of this study is that the individuals with ADHD participated in a 10-week remote monitoring period before this discussion, so they had direct experience of using remote monitoring measures, including smartphone apps and wearable devices. We still introduced a hypothetical scenario of longer future RMT studies, as this is beneficial to gain insight into what participants would want from future research. Future research should complete debrief interviews after participants have completed longer RMT studies to understand whether barriers to and facilitators of RMT changes when participating for a year or two. We have already incorporated this consideration into our “ADHD Remote Technology Study of Cardiometabolic Risk Factors and Medication Adherence” study, where individuals with ADHD will be measured for 12 months and will provide detailed qualitative feedback on their participation [27].

The gender imbalance in this study is also a limitation that should be acknowledged. In adulthood, the prevalence rate of ADHD is similar for male individuals and female individuals [30]; however, in this study, only 3 (two participants with ADHD and 1 participant in the comparison group) of 22 participants who completed the interviews were male. It could be that female individuals have views on RMT that may not be shared with male individuals. Owing to the imbalance of genders, we were unable to compare the views between female individuals and male individuals; this is an important topic for future research.

### Conclusions

This study builds on emerging research on the barriers to and facilitators of using RMT in psychiatric disorders in health care and long-term monitoring. We have shown the barriers to and facilitators of using RMT in people with ADHD following a 10-week remote monitoring study period. The themes that emerged during this qualitative analysis suggest that people with ADHD agree that RMT can provide useful objective data that uses repeated measurements with ongoing active (eg, questionnaires) and passive (eg, smartphone sensors and wearable devices) monitoring. Although themes overlapped with previous research on barriers and facilitators of engagement with RMT (eg, depression and epilepsy) and with a comparison group, there are unique considerations for people with ADHD, for example, understanding the impact of ADHD symptoms has with using RMT and reducing the frequency of cognitive task administration. Researchers need to continue to work with people with ADHD to develop future RMT studies for longer periods.

## Appendix

Multimedia Appendix 1 Final major, minor, and subthemes of barriers to and facilitators of remote measurement technology that emerged across participant groups.

## Abbreviations

ADHD: attention-deficit/hyperactivity disorder
ART: ADHD Remote Technology
GP: general practitioner
RMT: remote measurement technology

## Data Availability

For open access, the author has applied a Creative Commons Attribution (CC BY) license to any author-accepted manuscript version. The data supporting this study are not openly available but may be shared on request following a formal review process by the chief investigator and coinvestigators.
