# Supplementary material for: Barriers to and Facilitators of Using Remote Measurement Technology in the Long-Term Monitoring of Individuals With ADHD: Interview Study
Source: JMIR Form Res. 2023 Jun 30;7:e44126. doi: 10.2196/44126 (PMC10365629; doi:10.2196/44126)
Supplement: Multimedia Appendix 1 [file formative_v7i1e44126_app1.docx]

**Multimedia Appendix 1.** Final major, minor, and subthemes of barriers to and facilitators of remote measurement technology that emerged across participant groups.

|  | | | | ADHD^a^ group (n=10), n (%) | Comparison group (n=12), n (%) | Total (n=22), n (%) |
| --- | --- | --- | --- | --- | --- | --- |
| **Health related** | | | | | | |
|  | **Insight** | | | | | |
|  |  | **Health-related behaviors** | | | | |
|  |  |  | Sleep | 9 (90) | 9 (75) | 18 (82) |
|  |  |  | Physical activity | 6 (60) | 8 (67) | 14 (64) |
|  |  |  | Improvements | 6 (60) | 10 (83) | 16 (73) |
|  |  | **Lifestyle** | | | | |
|  |  |  | Phone usage | 2 (20) | 1 (8) | 3 (14) |
|  |  |  | Routine | 1 (10) | 0 (0) | 1 (5) |
|  |  |  | Improvements | 3 (30) | 0 (0) | 3 (14) |
|  | **Impact of ADHD symptoms** | | | 10 (100) | 1 (8) | 11 (50) |
|  |  | Difficulties with attention | | 6 (60) | 0 (0) | 6 (27) |
|  |  | Disorganization | | 4 (40) | 0 (0) | 4 (18) |
|  |  | Forgetfulness | | 6 (60) | 1 (8) | 7 (32) |
|  |  | Interpretation | | 1 (10) | 0 (0) | 1 (5) |
|  |  | Motivation for demanding tasks | | 4 (40) | 0 (0) | 4 (18) |
|  |  | Restlessness | | 1 (10) | 0 (0) | 1 (5) |
| **User related** | | | | | | |
|  | **Perceived costs** | | | | | |
|  |  | **Active monitoring** | | | | |
|  |  |  | Cognitive tasks | 6 (60) | 2 (17) | 8 (36) |
|  |  |  | Questionnaires | 1 (10) | 1 (8) | 2 (9) |
|  |  | **Passive monitoring** | | | | |
|  |  |  | Charging devices | 2 (20) | 1 (8) | 3 (14) |
|  |  |  | Wearing Fitbit | 1 (10) | 0 (0) | 1 (5) |
|  |  |  | Adapting to new study phone | 3 (30) | 1 (8) | 4 (18) |
|  |  |  | Connectivity | 2 (20) | 1 (8) | 3 (14) |
|  | **Compatibility** | | | | | |
|  |  | Fit into their daily life | | 10 (100) | 12 (100) | 22 (100) |
|  |  | Some difficulty finding the time | | 4 (40) | 3 (25) | 7 (32) |
|  | **Intrinsic value** | | | | | |
|  |  | Benefits of participating outweighed the costs | | 9 (90) | 12 (100) | 21 (95) |
|  | **Technology acceptance** | | | | | |
|  |  | Passive data | | 10 (100) | 12 (100) | 22 (100) |
|  |  | Technology nowadays collects these data | | 3 (30) | 5 (42) | 8 (36) |
|  |  | Well explained | | 2 (20) | 4 (33) | 6 (27) |
|  |  | Trust | | 5 (50) | 4 (33) | 9 (41) |
|  | **Overall experience** | | | | | |
|  |  | **Positive** | | | | |
|  |  |  | Easy | 7 (70) | 9 (75) | 16 (73) |
|  |  |  | Enjoyable | 8 (80) | 10 (83) | 18 (82) |
|  |  |  | Interesting | 4 (40) | 5 (42) | 9 (41) |
|  |  |  | Study period went quickly | 3 (30) | 3 (25) | 6 (27) |
|  |  |  | Well organized | 2 (20) | 2 (17) | 4 (18) |
|  |  | **Negative** | | | | |
|  |  |  | Adjustment period | 1 (10) | 2 (17) | 3 (14) |
|  |  |  | Demanding | 1 (10) | 1 (8) | 2 (9) |
|  |  |  | Long | 0 (0) | 3 (25) | 3 (14) |
| **Technology related** | | | | | | |
|  | **Value in gathering** **RMT^b^** **data** | | | 9 (90) | 12 (100) | 21 (95) |
|  |  | Objective measures of your health | | 6 (60) | 5 (42) | 11 (50) |
|  |  | Better than manually providing the data | | 3 (30) | 8 (67) | 11 (50) |
|  | **Convenience** | | | | | |
|  |  | **Passive monitoring** | | | | |
|  |  |  | Made it easier | 5 (50) | 7 (58) | 12 (55) |
|  | **Intrusiveness** | | | | | |
|  |  | **Cognitive tasks** | | | | |
|  |  |  | Tedious | 10 (100) | 9 (75) | 19 (86) |
|  |  |  | Frequency of administration | 4 (40) | 4 (33) | 8 (36) |
|  |  |  | Too long | 6 (60) | 5 (42) | 11 (50) |
|  |  | Concerns of monitoring | | 3 (30) | 3 (25) | 1 (27) |
|  |  | Aggression questionnaire | | 2 (20) | 1 (8) | 3 (14) |
|  | **Usability** | | | | | |
|  |  | **Wearable device (Fitbit)** | | | | |
|  |  |  | Comfort | 7 (70) | 8 (67) | 15 (68) |
|  |  |  | Compliance | 8 (80) | 11 (92) | 19 (86) |
|  |  |  | Technical issues | 6 (60) | 8 (67) | 14 (64) |
|  |  | **Study Android phone** | | | | |
|  |  |  | Easy to use | 4 (40) | 4 (33) | 8 (36) |
|  |  |  | Technical challenges | 9 (90) | 5 (42) | 14 (64) |
|  |  |  | Used phone as normal | 4 (40) | 5 (42) | 9 (41) |
|  |  | **Active App** | | | | |
|  |  |  | Easy to complete | 6 (60) | 7 (58) | 13 (59) |

^a^ADHD: attention-deficit/hyperactivity disorder.

^b^RMT: remote measurement technology.

Percentages are used to compare the themes that deviated between individuals with ADHD and individuals without ADHD in a comparison group.
